# Building capacity for dissemination and implementation to maximize research impact in a CTSA: The University of Wisconsin story

**DOI:** 10.1017/cts.2020.3

**Published:** 2020-01-10

**Authors:** Andrew Quanbeck, Jane Mahoney, Kim Kies, Kate Judge, Maureen Smith

**Affiliations:** 1 Department of Family Medicine and Community Health and Health Innovation Program, University of Wisconsin–Madison, School of Medicine and Public Health, Madison, WI, USA; 2 Department of Medicine, University of Wisconsin–Madison, School of Medicine and Public Health, Madison, WI, USA; 3 Dissemination & Implementation Launchpad, Institute for Clinical and Translational Science, School of Medicine and Public Health, Madison, WI, USA; 4 Community-Academic Partnership Program, Institute for Clinical and Translational Science, School of Medicine and Public Health, Madison, WI, USA; 5 Department of Population Health Sciences, Department of Family Medicine and Community Health, and Health Innovation Program, University of Wisconsin–Madison, School of Medicine and Public Health, Madison, WI, USA

**Keywords:** Evidence-based practice, implementation, dissemination, capacity building, designing for dissemination

## Abstract

We report results of an 8-year process of stakeholder engagement aimed at building capacity in Dissemination and Implementation (D&I) research at the University of Wisconsin as part of the National Institutes of Health’s Clinical and Translational Science Award (CTSA). Starting in 2008, annual individual interviews were held with leaders of the Wisconsin CTSA’s community engagement core for strategic planning purposes. Interviews were followed by annual planning meetings that employed a facilitated group decision-making process aimed at identifying and prioritizing gaps in the translational research spectrum. In 2011, the stakeholder engagement process identified D&I as a primary gap limiting overall impact of the institution’s research across the translational spectrum. Since that time, our CTSA has created an array of D&I resources falling into four broad categories: (1) relationship building with D&I partners, (2) D&I skill building, (3) translational research resources, and (4) resources to support D&I activities. Our systematic process of stakeholder engagement has increased the impact of research by providing D&I resources to meet investigator and community needs. CTSAs could engage with leaders of their community engagement cores, which are common to all CTSAs, to adapt or adopt these resources to build D&I capacity.

## Introduction

The University of Wisconsin–Madison (UW) perennially ranks among the nation’s preeminent research institutions, placing sixth in 2017 with research funding of $1.2 billion [1]. UW is home to the Institute for Clinical and Translational Research (ICTR), one of the nation’s 61 federally awarded Clinical and Translational Science Award (CTSA) grantees. The university is a land grant institution guided by the “Wisconsin Idea,” which holds that the university’s research should be applied to improve the health and quality of life for citizens of the state and the nation.

The Wisconsin Idea has guided ICTR in aiming to improve the health of the communities it serves by encouraging the dissemination of UW research. A national survey of 266 public health researchers across the USA revealed wide variability in how researchers incorporate the concept of dissemination into their research [2]. This environmental scan, including investigators from many CTSAs and government agencies, suggested considerable room for improvement in designing for dissemination; 73% of respondents estimated they spent less than 10% of their time on dissemination, and only about one-third of respondents always or usually involved stakeholders in the research process. Findings of the survey raise important questions about how research institutions can promote dissemination and implementation (D&I) and motivate researchers to place more emphasis on translating research findings into practice.

The purpose of this article is to describe how CTSA leaders at UW built a comprehensive system designed to improve the health of communities statewide by incorporating D&I concepts across the translational research spectrum. Before telling this story, it is worth considering the question: Why should a CTSA and its leadership invest in D&I? After all, CTSA funding is not conditional on any mandated D&I activities. The story reported here spans 8 years and represents a significant outlay of resources. We tell the story in hopes that other CTSA leaders may find UW’s experience instructive in deciding whether to invest in D&I capacity at their own institutions and to provide practical guidance on building D&I capacity for those who choose to do so.

## Methods

Our story begins in 2007, with the granting of UW’s inaugural CTSA and the establishment of ICTR’s Community–Academic Partnerships (CAP) core. ICTR-CAP is a federation of 38 community- and practice-based research networks and academic programs distributed across the university and the state. ICTR-CAP program partners receive funding or other incentives to offer resources that support the goals of the CAP core and are expected to engage in annual strategic planning by attending ICTR-CAP steering committee meetings (4–6 times per year), meeting with the ICTR-CAP Director annually to review progress and plan for the upcoming year, and submitting performance metrics twice per year.

The 2011 strategic planning process had a goal of exploring gaps in the translational research spectrum at UW to inform planning for ICTR’s second round of renewal, submitted in 2012. As part of this process, the ICTR-CAP Director (co-author Smith) met with the ICTR-CAP steering committee (composed of leaders from each of the networks and programs in the federation), followed by individual interviews with each member. These interviews were followed by a strategic planning meeting that employed a facilitated group decision-making process to identify and prioritize gaps in the translational research spectrum.

**Fig. 1.**
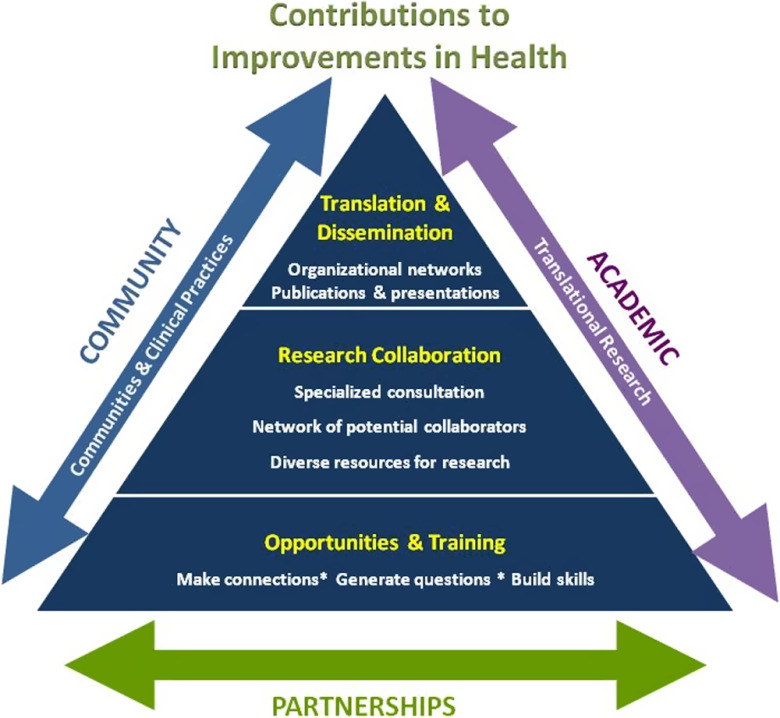
Results of 2011 strategic planning process.

The strategic planning process conducted in 2011 developed a schematic to characterize the resources provided by ICTR-CAP and identified a lack of resources for D&I as the primary gap limiting the overall impact of the science derived from the institution’s research across the translational spectrum. This gap is denoted in the top level of the pyramid depicted in Fig. 1. The gap analysis illustrated that while the University has well established systems in place to support the dissemination of patentable technologies (e.g., drugs and devices), no such infrastructure existed to support the dissemination of non-patentable innovations. Further, few investigators engaged in D&I research within the CTSA.

The ICTR-CAP Director and ICTR-CAP program leaders committed to systematically improve the resources available for D&I over the following 8 years. The institution’s commitment to building D&I capacity was explicitly included in our 2012 CTSA renewal application as one of the three aims of the community engagement core, specifically to: “Increase the use of research to improve practices, programs, and policies; disseminate research results and methodologies through existing community, practice, and policy networks; and facilitate D&I of research results between investigators and their community partners.” The stakeholder engagement and strategic planning process have provided a method to continuously identify gaps and opportunities in D&I resources via annual reassessments.

**Fig. 2.**
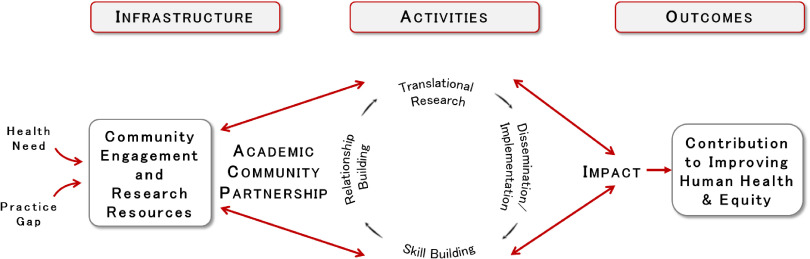
Conceptual model for capacity building.

A new ICTR-CAP conceptual model was developed for our 2012 CTSA renewal application that specifically incorporated D&I. Fig. 2 illustrates the conceptual approach used and the activities that resulted from iterated application of the stakeholder engagement process. This iterative process of inquiry and action led to activities that fell into the four general categories shown in the center of the figure. We will use these four activities as the basis for categorizing our D&I resources.

*Relationship building* includes functions such as matching investigators to academic and community resources, including access to networks and organizational research partners (such as practice-based research networks). *Translational research resources* include CTSA-based grant funding and providing access to skilled researchers who can advise investigators in pursuing extramural funding. *Skill building* pertains to providing training in skills germane to D&I research, including short courses and videos accessible to the public. *Dissemination and implementation resources* include support for investigators in packaging their interventions for scale-up, dissemination, and commercialization.

## Results

A summary of D&I gaps identified and specific strategies to address these D&I gaps is detailed in Table 1. Entries in the table are presented in chronological order. Tools used to support these D&I strategies are available at www.hipxchange.org/DevelopD&I. Launched in 2013 after our CTSA was renewed, these strategies built on the existing infrastructure for the ICTR-CAP core, which included (a) community- and practice-based research networks for primary care, health systems, aging, pharmacy, and public health, (b) a pilot awards program that has supported 92 pilots with a total of $7.3 million, (c) an external community stakeholder committee that makes final recommendations for pilot funding, (d) an educational core that trains investigators, staff, and students in community and stakeholder-engaged research methods, including community-based participatory research and qualitative/mixed methods [3], and (e) research consultation and collaboration services that provide access to experienced researchers across more than 25 disciplines through partnerships with academic programs distributed across the university. Tools used by the ICTR-CAP core to support this broad spectrum of activities are available at www.hipxchange.org/BuildingaCERCore.

**Table 1.** Results of ICTR-CAP stakeholder engagement process to build D&I capacity

Year (began)	Gap	Strategy	Outcomes to date	Activity/stream
2013	D&I work needed an organizing home within ICTR-CAP	Create the D&I Research Resource (later renamed the D&I Launchpad).	D&I Launchpad includes two part-time faculty and three full-time academic staff	D&I resources, translational research resources
2013	Researchers across campus were generally unfamiliar with D&I science.	Create D&I Short Course	318+ short course attendance	Skill building
2013	Researchers lack funding to research how to implement proven innovations.	Create the D&I Research Pilot Award program	13 funded	Translational research resources
			~$1,949,800	
2013	Researchers lack support needed to effectively translate innovations into practice.	Create the ICTR-CAP Dissemination Supplement Award	9 funded	Translational research resources
			~$10 k each	
2013	Researchers had no central place or central support to document or package innovations for dissemination.	Create a web portal (HIPxChange) and provide support to document and disseminate materials and develop trainings	52 toolkits	D&I resources
			>11,000 downloads	
2014	Campus researchers needed support and resources to submit competitive research grants.	Create the D&I consultation service.	503 consults	Translational research resources
2014	Clinical researchers lacked knowledge of how to engage stakeholders at early stages of the translational research spectrum to maximize D&I potential.	Add D&I content to the ICTR-CAP Stakeholder and Patient Engagement short courses and tools.	8 toolkits, online video training	Skill building
			143 attend short courses	
2017	Researchers face challenges scaling up innovations to the point of commercialization.	Create the ICTR-CAP Evidence-to-Implementation Award	2 awards made in 2018; second round rolled out in 2019	D&I resources
2017	Investigators need incentives to plan for dissemination early on	Redesign ICTR-CAP pilot grants to require a dissemination plan.	11 awards have dissemination plans	Translational research resources
2019	Investigators need incentives to engage adopters in designing interventions.	Redesign ICTR-CAP pilot grants to require engagement of an adopter during the design of an intervention.	Forthcoming	Translational research resources
2019	Researchers lack easy access to adopters of interventions.	Engage community- and practice-based research networks in identifying adopters to participate in design for dissemination activities	Forthcoming	Relationship building
2019	Scaling an intervention often requires a working with a purveyor organization	Create the ICTR-CAP Dissemination Pipeline Award to partner ICTR-CAP researchers and interventions with purveyor organizations	Forthcoming	D&I resources

D&I, Dissemination & Implementation; ICTR, Institute for Clinical & Translational Research; CAP, Community–Academic Partnership; HIP, Health Innovation Program.

*Gap*: D&I work needed an organizing home within ICTR-CAP.

*Response*: The D&I Research Resource (subsequently renamed the D&I Launchpad) was created in 2013, convening a core group of faculty and staff to provide support for investigators campuswide in conducting translational research across the spectrum. The D&I Launchpad is led by co-author Mahoney and employs one part-time faculty member (co-author Quanbeck), one full-time PhD-level research scientist (co-author Kies), and three full-time academic staff members (as of 2019). Support provided by the D&I Launchpad began with scientific advising (process and outcomes evaluation and D&I research methods) and eventually moved to encompass intervention packaging (e.g., developing and beta-testing implementation guides and other implementation support tools) and business strategies (marketing, pricing, and sales forecasting).

*Gap*: Researchers across campus were generally unfamiliar with D&I science.

*Response*: The annual D&I Short Course was created for researchers and community stakeholders to explore this emerging field with national and local experts, teaching the basics of D&I to researchers so they can effectively spread their research. Themes vary annually and have included health equity in D&I research, choosing D&I frameworks, and D&I study design. Attendance has grown steadily since inception, averaging more than 80 attendees from 2016 to 2018. Increasingly, attendance has come from researchers across the USA. Guest faculty have included some of the most influential scholars in the D&I field.

*Gap*: Researchers lacked funding to research how to implement proven innovations.

*Response*: The D&I Research Pilot Award program was created to support research on how to implement evidence-based interventions. (The ICTR Pilot Award program, mentioned above, addresses the development of innovations, while this program specifically addresses research on *dissemination and implementation*.) The awards provide up to $150,000 for 18 months. Awardees’ D&I research has focused on identifying strategies and methods to maximize outcomes including reach, uptake, feasibility, fidelity, acceptability, maintenance, and scale-up of evidence-based interventions. Applicants are expected to use the results of the D&I pilot award to apply for federal funding.

*Gap*: Researchers lack support needed to effectively translate innovations into practice.

*Response*: The ICTR Dissemination Supplement Award was created to provide funding for the creation of materials necessary to disseminate interventions developed at UW. Nine awards were made between 2014 and 2016. The program was eventually transitioned into the Evidence-to-Implementation Award, described later.

*Gap*: Researchers had no central place to document or package innovations for dissemination.

*Response*: UW’s Health Innovation Program supports research projects in collaboration with local and statewide health care systems and serves as an academic home for junior faculty interested in D&I research careers. The UW’s Health Innovation Program created and supports “HIPxChange,” a website offering toolkits that promote the implementation and dissemination of innovations developed by UW researchers. Open to the general public, HIPxChange hosts 52 toolkits with more than 11,000 downloads as of 2019.

*Gap*: Campus researchers need support to submit competitive D&I research grants.

*Response*: The D&I Launchpad launched a consult service to support high-quality grant applications for both D&I-focused grant submissions and grants with D&I components. The consult service assists research teams apply D&I frameworks and design D&I studies. Since its inception, the D&I consult service has met with 503 researchers across the campus and the community, leading to the submission of 168 unique grants. Of these, 88 were approved – 26 federal grants, 45 CTSA-funded grants, and 17 external or institutionally based grants.

*Gap*: Researchers lacked knowledge on effectively engaging stakeholders in early stages of the translational research spectrum to maximize D&I potential.

*Response*: We added D&I content to the ICTR-CAP Stakeholder and Patient Engagement short courses and toolkits for promoting stakeholder engagement. The Stakeholder and Patient Engagement short courses provide experiential and didactic activities to develop skills in engaging stakeholders in research. Stakeholder engagement tools and consultations help to engage stakeholders in intervention design to enhance the feasibility of D&I.

*Gap*: Researchers face challenges scaling up innovations to the point of commercialization.

*Response*: The Evidence-to-Implementation Award was established to expedite the transfer and commercialization of evidence-based practices to end-users using resources from the D&I Launchpad. This award supports the creation of a D&I support package that includes a business plan, market research, pricing strategies, a value proposition, toolkits, checklists, training and promotional materials, health education materials, and evaluation tools. The award is intended to bridge the gap between D&I research and entrepreneurship.

Review criteria for the Evidence-to-Implementation Award were derived from an evidence-based predictive tool from the entrepreneurial literature designed to assess the commercial viability of innovations [4]. The tool demonstrated 98.8% specificity in predicting commercial failure. The review process was designed to maximize the probability that award funding is invested in projects that are practical and impactful. Review criteria include six dimensions: (1) features and benefits of the innovation, (2) demand for the innovation, (3) barriers to entry, (4) potential impact, (5) sustainability potential, and (6) readiness (of both the innovation itself, and of the investigators to bring the innovation to scale). A panel of seven expert reviewers scored applications using a process loosely based on NIH procedures.

We had seven applicants in the first year and issued two awards worth $75,000 each for 18 months. One project scales up an evidence-based Tai Chi program for falls prevention among older adults. The second award supports an academy for surgical coaching designed to promote knowledge transfer and skill building among surgeons. Though in a pilot stage, repeated iterations of the award will result in a longitudinal database that can be examined to determine whether the review criteria can prospectively assess the commercial success of evidence-based interventions.

*Gap*: Investigators need incentives to plan for dissemination at an early stage.

*Response*: In 2017, we redesigned the ICTR-CAP Pilot Award Program so that the request for proposals now explicitly requires that applicants articulate a dissemination plan for their research. Consultation with the D&I consult service is encouraged, and over half of pilot applicants have utilized D&I consultations to help plan for dissemination.

*Gap*: Investigators need incentives to engage adopters in designing interventions (“design for dissemination”).

*Response*: Although the ICTR-CAP pilot awards have always required a community stakeholder as a partner, we did not require this partner to be a decision-maker who could potentially decide to adopt the intervention at their organization. In 2019, we are further redesigning the ICTR-CAP Pilot Award Program so that the request for proposals now explicitly encourages applicants to engage with a potential adopter at the design stage. We are building toolkits for investigators to use with ICTR-CAP partners to enhance their knowledge of the “design for dissemination” concept and operationalize it in intervention design and pilot testing.

*Gap*: Researchers lack access to adopters of interventions.

*Response*: We are building on our robust set of community- and practice-based research networks to help investigators access to decision-makers who represent the stakeholders who would adopt proven interventions. These networks will connect investigators with adopters who can partner in intervention design to ensure feasibility up front—a key element of designing for implementation.

*Gap*: Scaling an intervention often requires working with a purveyor organization

*Response*: The D&I literature increasingly recognizes the essential role that purveyor organizations play in promoting the translation of research to practice [5,6]. In 2019, we created an award program called the “Dissemination Pipeline” to facilitate connections between investigators and purveyor organizations specializing in dissemination. The Dissemination Pipeline uses a systematic approach to promote dissemination, beginning with a call for proposals in which we ask investigators to demonstrate their interest in disseminating innovations they have developed. Through a rigorous application development process, we help investigators describe metrics of readiness for dissemination. We then approach existing purveyors to identify their interest in disseminating the innovation based on their knowledge of customer demand. Because purveyor organizations may have limited resources, the D&I Launchpad also provides support to purveyor organizations in packaging interventions.

## Determining Whether Components Are Working

Throughout our engagement process, we have evaluated the impact of every component created to address gaps. We start new initiatives with the idea of impact in mind, partnering with each ICTR-CAP program to brainstorm D&I metrics and creating systems to collect relevant data. Our mentality is that merely being proponents of D&I is insufficient—we must demonstrate value by moving the needle on important outcomes. For instance, a Tai Chi intervention for falls prevention was shown effective in a trial of 206 older adults conducted at 2 practice-based research networks in Wisconsin. The success of this effort led to a subsequent D&I Research Pilot Award in which the intervention was scaled up in a pragmatic randomized trial in community centers, in which the intervention was shown to improve leg strength, tandem balance, mobility and gait, balance confidence, and executive function. The program then received an Evidence-to-Implementation Award to create a sustainable business model for scaling the program to eight new adopter organizations statewide. During the period of the award (2018–2019), the adopter organizations supported training of 10 new instructors, who provided 6 Tai Chi Prime classes to 90 participants. As a condition of the award, investigators are expected to provide data on scale-up of the program for 3 years beyond the award’s end-date to provide a comprehensive picture of overall impact.

Measurement also helps us understand how well we have implemented changes to our system, and whether our solutions closed the gaps they were meant to address. Redesigning a CTSA’s data collection to reflect D&I and its role in improving health was an enormous effort. Beyond quantitative data collection (e.g., where innovations are implemented, number and demographic information on patients impacted), our efforts include eliciting impact stories—narrative pieces that lay out the value of D&I in a compelling and personalized way. These impact stories have been critical tools in communicating the value of D&I to institutional leaders and other stakeholders.

## Synthesis

**Fig. 3.**
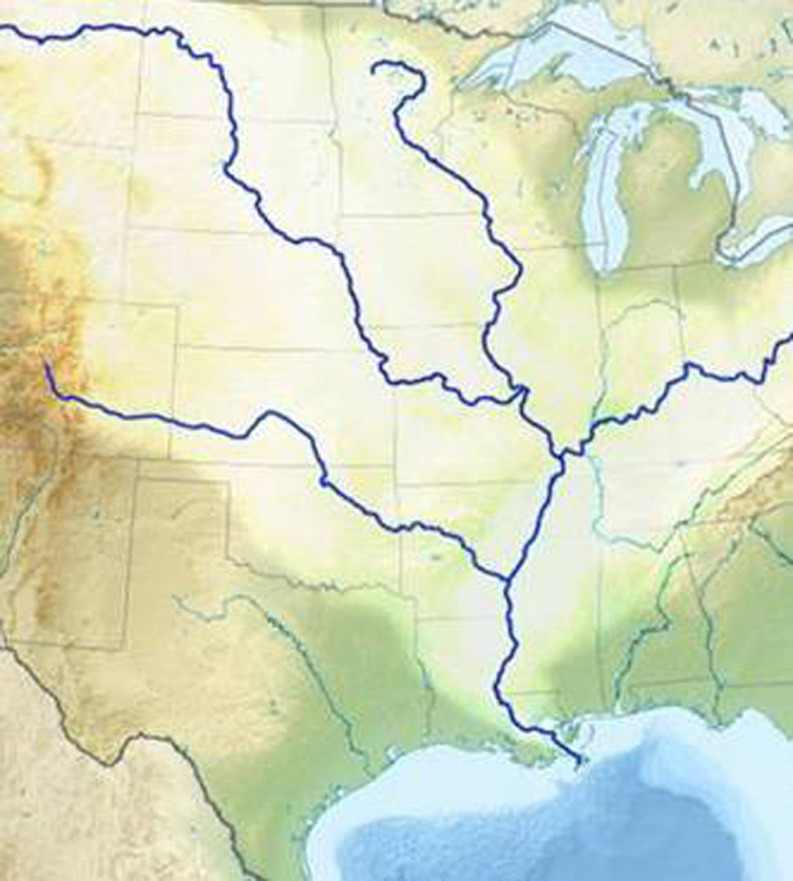
The Mississippi River System as an analogy for dissemination and implementation capacity building.

The Mississippi River (the world’s second largest river system) forms Wisconsin’s western border with neighboring states Minnesota and Iowa. At UW, we visualize our efforts at D&I capacity building using a river system analogy. The mighty Mississippi is fed by its main tributaries as it flows from north to south, growing in volume and force before emptying into the Gulf of Mexico. Similarly, we envision our efforts at relationship building with D&I partners, D&I skill building, creating D&I research resources, and support for direct D&I activities as streams contributing to a river of impact affecting the health and quality of life of the citizenry (see Fig. 3).

## Discussion

Beginning in 2011, stakeholders from the UW CTSA’s community engagement core conveyed that D&I was an essential missing component of our approach to translational research. This paper illustrates how we responded to that feedback to maximize the impact of translational research conducted at our CTSA. Although D&I as a discipline has grown substantially in size and influence over the past decade [7], CTSAs nationally have varied in their ability to incorporate D&I [8]. Collectively, it seems likely that CTSAs will face increasing demands to make D&I part of their mission, given the importance of demonstrating overall impact.

This paper shares one roadmap for CTSA leaders interested in building D&I capacity. Community engagement cores are common to all CTSAs and provide ready opportunities to partner with organizations that are motivated to offer the most effective evidence-based interventions available to serve their patients and communities. Based on our experience, we can offer five lessons learned:

1. *Goal orientation*. Building D&I capacity across the translational research spectrum requires vision and planning. Our vision was instantiated in the Specific Aims of our CTSA renewal applications and budgets, and those aims have driven our work on a day-to-day basis.
2. *Building D&I capacity is an iterative, incremental process*. We were able to build substantial resources through an iterative process. As each additional step succeeded, we identified additional gaps that needed to be filled. This suggests that CTSAs can accomplish D&I capacity building over time by starting small, pilot testing, and building incrementally based on the gaps identified through stakeholder engagement.
3. *Build in designing for dissemination early (and throughout) the translational spectrum*. D&I should not be an afterthought, even for researchers on the basic science end of the translational spectrum. As we have gained more experience, we identified the critical importance of encouraging (and increasingly requiring) intervention developers to solicit stakeholder input (including adopters) before, during, and after the development phase to maximize impact.
4. *Role of leadership*. Strong champions are needed in the CTSA to make this work. We benefitted from extraordinary institutional leaders who, though they were scientists but not D&I researchers *per se*, believed deeply in the value of D&I and its contribution to the impact of research conducted at the university. Literature on diffusion of innovations has been instructive [9,10]. Key leaders at the institution turned out to be early adopters with respect to the value of D&I. Advocating for an increased role for D&I at our institution required persistently telling stories that highlight the importance of maximizing the impact of an institution’s research and highlighting the role of D&I in this process.
5. *Matching funding is helpful*. Our CTSA has leveraged nonprofit foundation funding to support the work of building D&I capacity. While foundation funds have been essential to support D&I staff and build a comprehensive program, many of the D&I Launchpad’s activities have built on resources that are already part of ICTR-CAP programs, utilizing networks in the federation and promoting the philosophy of “design for dissemination” among them.

## Limitations and Challenges

At UW (and other CTSAs), we face institutional barriers that are fundamentally challenging. Pursuing tenure implicitly discourages D&I activities because the requirements make packaging and dissemination ancillary to research. The university in general does not have a business orientation. Researchers confront the reality that in the US health care system, transitioning toward sustainable business models (while largely outside of researchers’ control) must occur for evidence-based interventions to maximize their reach. Investigators who take interventions to commercialization face conflict-of-interest rules that, while necessary, can impede their ability to conduct D&I activities. Striking the right balance between D&I research and entrepreneurism is an emerging issue that federal agencies and CTSAs are increasingly contending with, as evidenced by examples such as the National Cancer Institute’s SPRINT training program (www.nci-sprint.com). Another important reality is that change within health care systems often outpaces research. While researchers may need a decade to take an innovation from a “good idea” to a proven one, health care systems continually face new problems that require implementation of solutions for which controlled trial data are often lacking.

## Conclusion

A growing literature stresses the importance of economic evaluation in implementation research and establishing a business case for D&I [11]. The return on investment in D&I can be measured by the impact of research conducted at our institution from the perspective of the communities the university serves. Through a concerted, long-term effort, we now have a system to guide projects from design and testing to publication and community impact. We have processes to assess projects at each stage of translation to evaluate their potential to move toward widespread dissemination, and, in some cases, commercialization. Many projects drop off at some point along the way, for a variety of reasons (e.g., innovations have efficacy but are not financially sustainable). Our system provides support as far along the translational spectrum as is feasible for each project.

Ultimately, we seek to build enthusiasm for D&I within CTSAs nationally. By sharing experiences, the field may collectively nudge [12] our way to an increased role for D&I within the broader CTSA landscape and demonstrate that the increased application of D&I increases impact.
